# Detection of hepatitis C virus in patients with terminal renal disease undergoing dialysis in southern Brazil: prevalence, risk factors, genotypes, and viral load dynamics in hemodialysis patients

**DOI:** 10.1186/s12985-015-0238-z

**Published:** 2015-02-03

**Authors:** Beatris Maria Vidales-Braz, Naylê Maria Oliveira da Silva, Rubens Lobato, Fabiana Nunes Germano, Luiza Dias da Mota, Elvino JG Barros, Ana Maria Barral de Martinez

**Affiliations:** Federal University of Rio Grande (FURG), Rio Grande, Brazil; Fluminense Federal University (UFF), Rio de Janeiro, Brazil; Federal University of Rio Grande do Sul (UFRGS), Porto alegre, Brazil

## Abstract

Hepatitis C (HCV) is a serious public health issue, and it is estimated that 3% of the world’s population is infected. Patients in hemodialysis units have an increased risk for contracting HCV, and high prevalence rates have been found in hemodialysis units around the world. This study is aimed at determining the prevalence of HCV in patients with terminal chronic renal disease (tCRD) who have been submitted to hemodialysis and peritoneal dialysis in southern Brazil to characterize the most prevalent genotypes, the viral load, and possible risk factors and to assess the validity between the ELISA and RT-PCR detection methods. Of 320 patients from three dialysis units, 318 participated in this study. According to the medical records, 55 patients were reactive to HCV, as determined via ELISA. All 318 samples were submitted to RT-PCR and genotyped using an Abbott Realtime® *m*2000 system. Data obtained through a questionnaire and chemical variables were associated with the HCV. Results: The prevalence of HCV was 18.24% (58), and the concordance between the HCV serology and the RT-PCR was 94%. Three patients were diagnosed to be negative for HCV using the ELISA assay but positive when using RT-PCR. Genotype 1 was the most prevalent (46.7%) genotype, within which subtype 1a was the most frequent (74.1%). One of the risk factors associated with HCV infection was the length of time that the patient had been undergoing hemodialysis treatments (p < 0.001). Additionally, the viral load was found to vary when tested before and after hemodialysis (p < 0.001). Conclusion: The prevalence of HCV in dialysis units continues to remain high, indicating nosocomial contamination. RT-PCR detected the presence of the hepatitis C virus in patients with a non-reactive serology, which highlights the importance of performing molecular tests on dialysis patients. The variation in the viral load in patients submitted to hemodialysis indicates a possible destruction or gripping of viral particles to the dialyzer membrane.

**Electronic supplementary material:**

The online version of this article (doi:10.1186/s12985-015-0238-z) contains supplementary material, which is available to authorized users.

## Introduction

In Brazil, between 2.5% and 4.9% of the population is infected with the hepatitis C virus (HCV). In the state of Rio Grande do Sul, the detection rate of 14.75 per 100 thousand inhabitants is considered high compared with the national average of 6.17 per 100 thousand inhabitants. This rate reflects the magnitude of the problem in the state [1-3].

Patients in dialysis units have been shown to have a higher risk for HCV infection compared with the prevalence of the antibody in groups of blood donors [3,4], which may contribute to the nosocomial dissemination in dialysis centers. There are several factors that are particularly related to the high prevalence rates, such as blood transfusions and the length of time that the patient has been undergoing hemodialysis [5,6].

According to the 2012 Census of the Brazilian Society of Nephrology, Brazil has an estimated 97,586 patients undergoing dialysis treatments, and the prevalence rate continues to increase [7]. Brazil is a continent-sized country, and although the prevalence of HCV among hemodialysis patients is known, with rates varying from 8.4% to 39.2%, the genotyping is not well documented [8]. Genotype 1 is predominant among hemodialyzed patients, and subtype 1a is the most frequently identified subtype, followed by 1b and 3a [8-10]. Two studies, one from Belo Horizonte, MG and a more recent study from Rio Grande/RS, showed that subtype 2b is the second most prevalent subtype [11,12].

The ELISA method (Enzyme-Linked Immunosorbent Assay), which is highly sensitive and specific, is used for the diagnosis and antibody screening of HCV in hemodialysis patients. However, patients with terminal chronic renal disease (tCRD) who are undergoing dialysis treatments may show a decrease in humoral and cellular immunity, which may lower the sensitivity of the test and give a false-negative result. For this reason, RNA-HCV detection using the RT-PCR technique (reverse transcription polymerase chain reaction) is necessary, as it detects small quantities of the virus in the bloodstream, dismissing any false-negative results and confirming the HCV diagnosis in these patients [13,14]. The analysis of the nucleotide sequence amplified using PCR followed by a phylogenetic analysis is the gold standard technique for detecting and identifying the genotypes and subtypes of HCV [15,16]. The most frequently used regions for sequencing are the 5′-UTR and the NS5b regions [14].

This study is aimed at determining, via RT-PCR, the prevalence of the hepatitis C virus among hemodialysis patients, the possible associated risk factors and the variation in the number of viral particles during hemodialysis in patients with tCRD from three dialysis units located in a municipality in southern Brazil.

## Patients and methods

### Target-population

The target population included patients who underwent dialysis in any of the three renal replacement therapy units in the city of Pelotas, RS, between March 2012 and August 2013. Of 320 patients, 318 signed the free and informed consent form, responded to a sociodemographic and behavioral questionnaire, and submitted a 5-milliliter sample of venous blood. Additionally, the biochemical variables from the same month were obtained from each interviewee’s medical record. The study was approved by the Health Research Ethics Committee, Plataforma Brasil on May 17, 2012 (http://aplicacao.saude.gov.br/plataformabrasil/login.jsf), with the decision number 23403 and the CAAE number 01902112.7.0000.5339.

### Detection, genotyping and the RNA-HCV viral load

A 5 mL sample of blood was collected before hemodialysis session using a vacuum tube containing EDTA (ethylenediaminetetraacetic acid) and was immediately centrifuged. A sample of blood was collected before and after hemodialysis session for identification of RNA-HCV viral load. The plasma was separated, and the sample was stored at -70°C until the analysis. The samples were submitted to molecular diagnosis (qualitative RNA-HCV test using RT-PCR) at the Molecular Biology Lab at FURG, Rio Grande. First, the viral RNA was extracted from a 140-μL sample of plasma using the QIAamp®Viral RNA kit (Qiagen lnc., Chatsworth, CA, USA). Following the extraction, the viral RNA was resuspended to a final volume of 10 μL in DEPC water containing 150 ng/μL of random primers (Life Technologies, Carlsbad, USA) and was incubated for 10 minutes at 70°C. The reverse transcription was carried out using 200 U of Moloney Murine Leukemia Virus Reverse Transcriptase (M-MLV RT) (Invitrogen), 0.1 M DTT, 25 U of RNaseOUT™ (Life Technologies) and 0.5 mM of each dideoxynucleotide (dATP, dTTP, dCTP, dGTP) for 90 minutes at 37°C in a thermocycler. To amplify a fragment from the 5′-UTR region of the HCV genome, the following initiators were used: NCR2 forward (5′-ATACTCGAGGTGCACGGTCTACGAGACCT-3′) and PTC1 reverse (5′-CGTTAGTATGAGTGTCGTGC-3′) for the first round of amplification and PTC3 forward (5′-GTGTCGTGCAGCCTCCAGG-3′) and NCR4 reverse (5′-CACTCTCGAGCACCCTATCAGGCAGT-3′) for the second round of amplification, according to Campiotto et al. [17]. The following initiators were used for NS5B region: PR3 (5′-TATGAYACCCGCTGYTTTGACTC-3′) and PR4 (5′-GCNGARTAYCTVGTCATAGCCTC-3′) for the first round of amplification and PR5 (5′-GCTAGTCATAGCCTCCGT-3′) and PR3 for the second round of amplification, according to Sandres-Sauné et al., [18]. A 225-bp fragment was obtained from the 5′-UTR region, and a 382-bp fragment was obtained from the NS5b region. The fragments were revealed under ultraviolet light after electrophoresis in a 2% agarose gel stained with ethidium bromide. Genotyping and viral load: the samples were genotyped using an Abbott Realtime® *m*2000 system in the CD4 and viral load lab of HU-FURG (Federal University of Rio Grande). Variables in the outcome: The independent variables that were analyzed were age, sex, level of education, complexion, marital status, blood transfusion, health care professional, use of injectable drugs, use of inhaled cocaine, sharing of syringes, surgical procedures, presence of tattoos or piercings, and presence of sexually transmitted diseases (STDs). The outcome or dependent variable was the presence of an HCV infection, confirmed via RT-PCR, the genotype and the viral load of each patient.

### Statistical analysis

The statistical analyses were carried out using STATA software, version 12.1 (StataCorp, Texas). In the descriptive analyses, percentages were used for the categorical variables; the mean and standard deviation were used for the symmetric quantitative variables; and the median and interquartile range were used for the asymmetric variables. In the bivariate analyses, a *χ*2 (chi-squared) test and Fischer’s exact test were used to test the differences in the categorical variables, and the *t*-test or the Wilcoxon test was used for the continuous variables. In the multivariate analyses, a backward Poisson regression was used to assess the independent effect of the variables, and the prevalence ratios and their respective robust 95% confidence intervals were calculated. The Wald test was used as a statistical test. The statistical significance cut-off point was p < 0.05.

## Results

Of the 318 patients who agreed to participate in this study, 287 (90.25%) were undergoing hemodialysis, and 31 (9.75%) were undergoing peritoneal dialysis. The 3 three surveyed clinics were referred to as Clinic 1, 2 or 3. All of the patients in “Clinic 1” came from the Single Health System (SUS), and 103 patients were interviewed. In “Clinic 2”, 100% of the patients were from the SUS, and 98 patients were interviewed. At “Clinic 3”, 90% of its patients came from the SUS, 10% came from private health plans, and 107 patients were interviewed. When looking at the types of renal replacement therapy (hemodialysis and peritoneal dialysis), 60% of the patients at “Clinic 1” were undergoing peritoneal dialysis, while “Clinic 2” had 3%, and “Clinic 3” had 4%. The prevalence of HCV in the patients from the three surveyed hemodialysis units was 18.24% (Clinic 1 had 18, Clinic 2 had 31 patients and Clinic 3 had 9 patients HCV-positive). Among those who had HCV, 51.7% were male, 55.2% did not have a partner, and their average age was 59.3 years (dp ± 16). The most prevalent group in terms of education level was the illiterate group (79.3%), and none of the HCV-reactive patients had a higher education degree. In terms of blood transfusion, 79.3% of the patients said they had received blood at least once in their lives. When asked about the use of condoms in different types of relationships (long-term or occasional partner), 65.5% of the patients said they had never used condoms. When analyzing the presence of the virus between the two types of dialysis, three categories emerged: category A: those who had always undergone hemodialysis; category B: those who had only undergone peritoneal dialysis; and category C: those who had undergone hemodialysis before starting peritoneal dialysis. Among all of the patients, 18.7% of the patients from category A and 8.6% from category C were found to be positive for HCV, while no patients from category B were found to have the virus.

In regards to the hemodialysis location, “Clinic 1” showed statistical significance in the gross analysis as well as the adjusted analysis (p < 0.001) and therefore offered a higher risk for HCV. The average duration of hemodialysis treatments among the 58 HCV-infected patients in this study was 101.6 months (dp ± 80.6), while the average duration for peritoneal dialysis was 5.4 months (dp ± 19.7). Based on the results of the model of logistic regression analyzing up the hemodialysis time, it is estimated that every year the HD patients has 6.7 times more likely to HCV infection. This shows a significant difference between the duration of therapy and the two types of dialysis treatments that were performed (p < 0.001) (Table 1).

**Table 1:** **Prevalence ratios and confidence intervals (CI 95**%**) between the presence of reactive HCV and the risk factors surveyed (n = 318)**

	**N**	**%**	**PR**	**CI 95** **%**	p-value*	**PR**	**CI 95** **%**	p-value*
Sex					0.559			0.458
Female	28	20.0	Ref. (1)	-		Ref. (1)	-	
Male	30	16.9	0.84	0.52; 1.34		0.84	0.53; 1.32	
Complexion					0.411			0.777
White	40	17.1	Ref.(1)	-		Ref. (1)	-	
Not white	18	21.4	1.25	0.76; 2.06		1.07	0.65; 1.81	
Age (years)					0.081			0.085
Up to 59	35	22.3	Ref. (1)	-		Ref. (1)	-	
60 or older	23	14.3	0,64	0.39; 1.03		0,66	0.41; 1.05	
Level of education					0.407			0.117
Illiterate	46	19.7	Ref. (1)	-		Ref. (1)	-	
Incomplete or complete Primary School	12	14.6	0.74	0.41; 1.33		0.71	0.46; 1.09	
Marital status					0.772			0.721
Without partner	32	19.1	Ref. (1)	-		Ref. (1)	-	
With partner	26	17.3	0.91	0.59; 1.45		0.91	0.57; 1.45	
Hospital					0,003^†^			0.001
Clinic 1	18	17.5	Ref. (1)	-		Ref. (1)	-	
Clinic 2	31	31.6	1.81	1.01; 3.24		1.54	0.90; 2.62	
Clinic 3	9	7.9	0.45	0.21; 0.98		0.44	0.20; 0.94	
HD e PD					0.063^†^			
Only HD	53	18.7	Ref. (1)	-		Ref. (1)	-	0.653
Only DP	-	-	-	-		-	-	
HD and then PD	5	27.8	1.48	0.59; 3.70		1.19	0.55; 2.58	

*PR: prevalence rate.

^†^Wald Test.

An adjusted analysis was performed by simultaneously adjusting the risk behaviors and the presence of STD. The prevalence of HBV among the patients was 2.6%, while HIV was 1%. A Poisson regression with a robust variance was also performed, and the presence of STD lost significance following the adjustment, but the co-infection with HBV remained significant (p < 0.006). The use of injectable drugs, a variable that was not significant in the gross analysis, became significant (p < 0.037) following the adjustment (Table 2).

**Table 2:** **Prevalence ratios and 95**% **confidence intervals between the presence of reactive HCV, the factors investigated and the behavioral data in the patients from Pelotas (n = 318)**

	**N**	**%**	**PR**	**CI 95** **%**	p-value*	**PR**_**A**_	**CI 95** **%**	**p-value**
Health Professional					0.712			-
No	55	18.0	Ref. (1)	-		-	-	
Yes	3	23.1	1.28	0.46; 3.55		-	-	
Blood transfusion					0.732			-
No	12	16.2	Ref.(1)	-		-	-	
Yes	46	18.9	1.16	0.65; 2.01		-	-	
Use of injectable drugs					0.153			0.037
No	56	17.8	Ref. (1)	-		Ref. (1)	-	
Yes	2	50.0	2.8	1.02; 7.69		4.16	1.08; 15.9	
Syringe sharing					0.998			0.587
No	52	18.4	Ref. (1)	-		Ref. (1)	-	
Yes	6	17.1	0.93	0.43; 2.00		0.76	0.31; 2.06	
Inhaled cocaine					0.998			0.381
No	57	18.3	Ref. (1)	-		Ref. (1)	-	
Yes	1	16.7	0.90	0.14; 5.53		0.51	0.12; 2.24	
Tattoo, piercing, acupuncture					0.438			0.300
No	12	18.8	Ref. (1)	-		Ref. (1)	-	
Yes	46	11.5	0.61	0.21; 1.82		0.56	0.18; 1.67	
HIV					0.332			0.184
No	57	18,1	Ref. (1)	-		Ref. (1)		
Yes	1	50.0	2.76	0.67; 9.23		2.35	0.85; 6.44	
HBV					0.006			<0.001
No	53	17.0	Ref. (1)	-		Ref. (1)		
Yes	5	62.5	3.65	2.02; 6.59		3.41	1.82; 6.38	
STD in the past year					0.049			0.098
No	45	16.4	Ref. (1)	-		Ref. (1)		
Yes	13	29.5	1.80	1.06; 3.05		1.48	0.83; 2.65	
Use of condom long-term partner					0.416^†^			0.452
Never	38	16.6	Ref. (1)	-		Ref. (1)		
Always	6	17.6	1.06	0.45; 2.51		1.08	0.34; 3.40	
Sometimes	14	25.5	1.53	0.83; 2.83		2.07	0.59; 7.17	
Use of condom occasional partner					0.673^†^			0.723
Never	38	16.9	Ref. (1)			Ref. (1)		
Always	6	20.0	1.18	0.50; 2.80		0.99	0.29; 3.37	
Sometimes	14	22.2	1.32	0.71; 2.43		0.66	0.19; 2.24	

*PR: prevalence rate.

^†^Wald Test.

The biochemical variables were obtained via medical records. The means and standard deviations of the symmetric variables were calculated according to the HCV results, and the p value indicates that the analyzed values are different between the HCV-positive and HCV-negative individuals. For the alanine transaminase (ALT), glucose and creatinine analysis, a non-parametric test (Wilcoxon) was performed for the asymmetric variables. For this reason, the description was made using the median value and the interquartile range (25^th^ and 75^th^ percentiles).

Among the samples sent for genotyping (n = 58), 81.03% (47) were genotyped because they were above the minimum detection limit (<12 IU/mL). Abbott®’s real-time HCV genotyping test detects genotypes 1, 2, 3, 4, 5, and 6 and subtypes a and b using a serum volume of 0.5 ml or 0.2 ml. The test’s sensitivity is ≥500 IU/mL, and its specificity is 97.0%. The quantification of RNA-HCV on the Abbott Realtime® *m*2000 system has a sensitivity of 12 IU/mL for a 0.5-ml sample, with a specificity of ≥99.5%. Genotype 1 was the most prevalent (46.7%), followed by genotype 3 (19.0%) and genotype 2 (5.3%). Genotypes 4, 5 and 6 were not identified. Within genotype 1, subtype 1a was the most the prevalent (74.1%), followed by subtype 1b (11.1%). In regards to the distribution of the genotypes among the hospitals, subtype 1a was the most prevalent in all three hospitals.

The viral load values were also analyzed, and statistically significant differences (p < 0.001) between the viral load values before and after the hemodialysis sessions were observed (Table 1). When the difference was analyzed by genotype, a significant difference (p < 0.001) was also observed between the viral loads (Table 3).

**Table 3:** **Viral load (number of copies of HCV-RNA per mL of plasma) for each HCV genotype in patients from in a city in southern Brazil (n = 58)**

			**Genotype**				
	**G1**		**G 2**		**G 3**		
	**Median**	**Pc 25 - Pc**	**Median**	**Pc 25 - Pc**	**Median**	**Pc 25 - Pc**	p-value*
	**(x1000)**	**75**	**(x1000)**	**75**	**(x1000)**	**75**	
Viral load before (IU/L)	467.1	165.2; 890.1	1,011.3	12.6; 3,018.1	267.9	25.8; 1,450	<0.001
Viral load after (IU/L)	376.3	12.9; 579.8	852.7	2.3; 1,676.8	1,001.3	1.7; 1,532.5	<0.001

*Only for patients undergoing hemodialysis treatment.

Among the studied individuals, three patients who had negative results according to the ELISA antibody survey showed positive RNA detection via the molecular tests, which shows 94% of concordance between the tests. RT-PCR has a higher sensitivity when compared to ELISA.

## Discussion

This is the first study to verify the prevalence, genotypes, viral load and risk factors connected with HCV infection in patients from dialysis units in the city of Pelotas in southern Brazil. The infection caused by this virus is still an ongoing problem in renal replacement therapy units. Currently, the hepatitis C virus is the most frequently found in patients with terminal chronic renal disease (tCRD) undergoing hemodialysis treatments. In Brazil, the number of patients undergoing dialysis treatments has been rapidly increasing, with an estimated 34,366 new cases every year [19], which includes an estimated 5,963 new cases in the southern part of the country [19]. These numbers highlight the grave problem that HCV represents in dialysis units, as dialysis units represent one of the main risk groups for HCV infection. The results show a continued high prevalence (18.24%) of HCV, confirmed via RT-PCR, even following the major advances in the techniques for detecting and preventing HCV, in addition to HCV education.

It is believed that the viral transmission may originate from several factors: surgeries and transfusions, to which the patients are often submitted, and biosafety factors, such as an unprepared technical team and the improper use of equipment for HCV-positive patients, which should be used exclusively by the infected patients. It may also be associated with the hemodialysis procedure itself (horizontal transmission), dermal transmission through aerosol or droplets during fistula cannulation, dialysis accidents involving blood spillage or transmission via contact with equipment used by contaminated patients [12,20,21]. When the relationship between the HCV positivity and the location of treatment was analyzed, the patients who received dialysis services at “Clinic 2” showed a 54% greater chance of being infected with HCV (p < 0.001). This situation was further confirmed by the adjusted analysis, revalidating the affirmation that this institution provides a potential risk for HCV infection to CRD patients. Further research is still necessary to corroborate this hypothesis.

In many developing countries, the primary method of infection is through blood transfusions because untested blood and blood derivatives are still used, in addition to poorly sterilized equipment. In Brazil, however, the implementation of triage tests in blood banks by the Ministry of Health in 1993, through directive n. 1376, along with the introduction of human recombinant erythropoietin, has led to a decrease in the transmission of the virus during transfusions [22,23]. In our study, blood transfusions prior to 1993 were significantly associated with the presence of HCV and were one of the most probable causes of infection reported by patients with HCV.

Another important factor in the transmission of HCV is surgery. One hundred percent of the HCV-positive patients had previously undergone some type of surgery. Surgery makes patients more prone to HCV infection because of the possible exposure to shared materials and the necessity for blood transfusion, especially prior to 1993 [24,25].

According to the literature data, HCV infection is associated with the number and duration of the hemodialysis treatments [24,26]. In this study, the length of time that the patient had been undergoing hemodialysis treatments was statistically significant for the HCV-positive patients (p < 0.001), with an average of 101.6 months (dp ± 80.6), indicating that the longer the hemodialysis permanence time the higher the probability of contracting HCV. It is not recommended for HCV patients to have isolation rooms, but there should be different technical teams to avoid any contact between HCV-positive and HCV-negative patients, as well as a only reusing HCV rooms for patients with hepatitis C and providing other rooms for patients with hepatitis B [27]. The movement of the nursing team and the sanitization team and the lack of isolation of HCV-positive patients are highly significant factors for the transmission of the hepatitis C virus.

In addition, it must be highlighted that the dialyzer should only be reused 12 times, according to directive n.82/GM (2000) from the Ministry of Health [4]. All three of the units that were assessed followed the rules of the National Health Surveillance Agency (ANVISA) RCD n.154 related to the reuse of dialyzers and to the attending of patients with reactive hepatitis B and C and HIV [28,29]. The patients and health professionals in dialysis units must be monitored so that it can be possible to determine the real risk factors for HCV infection. To prevent infection, it is necessary to implement quality biosafety programs in the units, ensuring that the workers are adequately trained, and a providing a supervisory technical team for epidemiological surveillance.

The peritoneal dialysis procedure is performed by the own patient in his home, with disposable equipment thus avoiding contact with infected patients, thus reducing the possible transmission, justifying our results of none patient infected with HCV in this modality.

The HCV detection method used in the surveyed dialysis units is the ELISA technique. This study made use of another molecular technique (RT-PCR) to investigate the prevalence of individuals infected by HCV. Therefore, we were able to compare the serological results with the detection of the viral RNA. Several authors have reported false-negative results in the serological tests of patients with tCRD undergoing renal replacement therapy. This highlights the importance of viral RNA identification in such patients [8,13,14,30]. The spread of HCV in dialysis units may be associated, among other aspects, with the difficulty in diagnosing the infection during the initial phase, when the serum conversion has not yet occurred [31]. This may have been a crucial factor for the lack of detection of the HCV antibodies in the individuals who tested positive for HCV via the RT-PCR methodology. In regards to the ELISA test, hemodialyzed patients may show false-negative results and unintentionally be exposed to possible nosocomial transmissions [13,32]. It is important to emphasize that the patients from renal replacement therapy units are considered to be immunosuppressed, which lowers the production of antibodies and consequently creates false-negative results in their tests. Therefore, the standard test used in these units should be viral RNA testing via PCR. The use of RT-PCR for the molecular diagnosis of HCV, using regions such as the 5′ UTR and NS5b from the viral genome, is a sensitive and specific methodology for the detection of the virus [14].

The distribution of these genotypes was similar to that found by Campiotto in a study performed using laboratory samples from different locations in Brazil, with genotype 1 being the most prevalent (64.9%), followed by genotypes 3 (30.6%) and 2 (4.6%). It is important to emphasize though that these samples did not solely belong to hemodialysis patients [17]. In a study performed with hemodialysis patients in the city of Goiás, Espirito-Santo identified genotypes 1a (65.7%), 1b (26.7%) and 3 (7.6%) to be the most prevalent [33]. In a study of blood donors from Distrito Federal, Amorim et al. identified the prevalence of genotypes 1a (82.3%), 3a (9.8%), and 1b (5.9%), followed by the co-infection of 1a/1b (2%) [10]. In our study, co-infections of the different genotypes were not identified. It has already been shown that genotype 1a is found in significant percentages in Brazil [10,33], although da Silva et al. have identified the prevalence of genotype 1b followed by genotype 2b [12]. Further studies will be performed throughout southern Rio Grande do Sul to assess the distribution of the genotypes in dialysis units in southern Brazil.

The viral load had a significant variation when tested before and after the hemodialysis session (p < 0.001). The average was 44×104 IU/L (dp: 64×104) before hemodialysis and 34×103 (dp: 50×104) after hemodialysis, indicating the possible destruction or adherence of the viral particles to the dialyzer membrane [34-36]. This may mean that patients undergoing hemodialysis have a more stable viral load when compared to those who are not undergoing this type of treatment [37,38]. It is well known that the HCV viral load influences the course of the antiviral treatment and is used to assess the virological response. Patients with hepatitis undergoing hemodialysis actually live longer, as there are studies showing that there is less hepatic damage [39,40]. It was observed that genotype 2 had the highest viral load, 11×105 (dp: 17×105), compared with genotypes 1 and 3. When analyzed after the hemodialysis session, genotype 3 showed an increase in the viral load from 69×104 (dp: 72×104) to 74×104 (dp: 71×104). These results indicate that more studies of the viral load in relation to hemodialysis treatments are necessary.

The average ALT level found among the patients from the units studied was of 15.3 IU/L (dp ± 12.3). When analyzing the values of this enzyme only in the group of patients with HCV, the average changed to 24.2 IU/L (dp ± 20.6), which is significantly higher (p < 0.001) than the average found by Fabrizi and contributors (18.9 IU/L), in their study correlating hepatic damage with aminotransferases and viral load during a 13-month period [34]. The mean ALT values increased when comparing the HCV-positive and HCV-negative patients, but the values were still within the “normal” levels (20 IU/L) [34,41]. Altered ALT levels are not common in patients with HCV undergoing hemodialysis treatments, which suggests that aminotransferases are not good predictors of hepatocellular injury for these patients because it is believed that there is enzymatic inhibition of the aminotransferases due to uremic toxins, pyridoxine deficiency (cofactor of aminotransferases), loss of enzymes in dialysis, interference of other dialyzable substances in the dosage of transaminase activity and lactate consumption of the factors that are necessary for the dosage of transaminase activity [39]. Several studies have shown that the ALT levels can be higher in HCV-positive patients compared with HCV-negative individuals, even when the values are below those considered to be normal, and the aminotransferase variations are related to the HCV viral load [34,42].

## Conclusion

The high prevalence of HCV in patients undergoing hemodialysis is still a grave problem. RT-PCR is proven to be more sensitive than the routine serological tests performed in the clinics studied, indicating that molecular tests are a better option for patients undergoing dialysis. Co-infection with HBV and the use of injectable drugs are risk factors for HCV infection, as well as the overall length of time that the patient has been undergoing hemodialysis treatments. The variation in the viral load when tested before and after hemodialysis showed different values when correlated to the different viral genotypes. These results show the importance of aiming new research on this area to assess and compare the reuse of dialyzers, and the results further indicate the importance of single-use dialyzers as a protection factor against HCV.
